# Analysis of risk factors for carotid intima-media thickness in patients with type 2 diabetes mellitus in Western China assessed by logistic regression combined with a decision tree model

**DOI:** 10.1186/s13098-020-0517-8

**Published:** 2020-01-28

**Authors:** Yuan-Yuan Zhou, Hong-Mei Qiu, Ying Yang, Yuan-Yuan Han

**Affiliations:** 1 Department of Endocrinology and Metabolism, Fourth Affiliated Hospital of Kunming Medical University, The Second People’s Hospital of Yunnan Province, Kunming, 650021 China; 2 Department of Endocrinology and Metabolism, Sixth Affiliated Hospital of Kunming Medical University, The People’s Hospital of Yuxi City, Yuxi, 653100 China; 3 Center of Tree Shrew Germplasm Resources, Institute of Medical Biology, Chinese Academy of Medical Sciences and Peking Union Medical College, Kunming, 650021 China

**Keywords:** Type 2 diabetes, Carotid intima-media thickness, Visceral fat area, Decision tree

## Abstract

**Background:**

Cardiovascular disease (CVD) is the leading cause of morbidity and mortality in patients with type 2 diabetes (T2DM). Carotid intima-media thickness (CIMT) is considered a preclinical stage of atherosclerosis. Therefore, it is necessary to identify the related risk factors for CIMT to facilitate the early prevention of CVD. Previous studies have shown that visceral fat area (VFA) is a risk factor for T2DM and CVD. However, few studies have focused on the effects of VFA on CIMT associated with T2DM. Moreover, considering that the body fat distribution shows regional and racial heterogeneity, the purpose of this study was to investigate the predictive value of VFA and other risk factors for CIMT associated with T2DM in Western China.

**Methods:**

In a cross-sectional study, a total of 1372 patients with T2DM were divided into the CIMT (−) group (n = 965) and the CIMT (+) group (n = 407) based on CIMT values. In addition to the univariate analyses, logistic regression analysis and a decision tree model were simultaneously performed to establish a correlation factor model for CIMT.

**Results:**

Univariate analyses showed that sex, smoking status, age, heart rate, systolic blood pressure (SBP), diastolic blood pressure (DBP), height, weight, body mass index (BMI), waist circumference, hip circumference, waist-hip ratio, VFA, subcutaneous fat area, and the levels of 2-h C-peptide, serum creatinine, urea nitrogen and uric acid were significantly different between the two groups (all *p *< 0.05). Smoking, increased VFA, female sex and increased BMI were risk factors in the logistic regression analyses (OR = 5.759, OR = 1.364, OR = 2.239, OR = 1.186, respectively). In the decision tree model, smoking was the root node, followed by sex, waist circumference, VFA and chronic kidney disease (CKD) in order of importance.

**Conclusions:**

In addition to smoking, sex and BMI, VFA has a significant effect on CIMT associated with T2DM in the Chinese Han population in Western China. In addition, the decision tree model could help clinicians make more effective decisions, with its simplicity and intuitiveness, making it worth promoting in future medical research.

*Trial registration* ChiCTR, ChiCTR1900027739. Registered 24 November 2019-Retrospectively registered, http://www.chictr.org.cn/index.aspx.

## Background

With continuous improvements in living standards, type 2 diabetes (T2DM) has become a serious threat to human health. According to International Diabetes Federation (IDF) statistics, in 2011, the global number of patients with T2DM reached 366 million people, of whom 80% are in developing countries. It is estimated that there will be nearly 552 million T2DM patients worldwide by 2030 [1]. The risk of cardiovascular disease (CVD) in patients with T2DM is 2–3 times higher than that in non-T2DM patients [2]. CVD is the leading cause of morbidity and mortality in patients with T2DM worldwide [3]. Therefore, it is very strategically important to prevent and treat diabetic macrovascular disease.

Cardiovascular disease (CVD) is a general term for heart and vascular diseases, such as acute coronary syndrome, myocardial infarction, stroke, and peripheral artery diseases [4–6]. Atherosclerosis is the underlying cause of most CVD [7]. More importantly, increased carotid intima-media thickness is considered as an early deterioration in the arterial intima and is a preclinical stage of atherosclerosis [8, 9]. Despite controversial results, the majority of studies have recommended measuring CIMT in clinical practice for the assessment of cardiovascular risk [10–15]. Therefore, it is necessary to identify the relevant risk factors for CIMT to facilitate the early comprehensive prevention and treatment of macroangiopathy. According to previous studies, CIMT can be affected by many factors, including age [16–18], sex [16–18], smoking [19, 20], blood pressure [21], blood lipid levels [22], thyroid function [23, 24], blood glucose levels [25], blood glucose level fluctuations, and C-peptide levels [26, 27].

Recently, research regarding the effects of fat metabolism on CVD has become a challenging topic globally. Recently, the International Atherosclerosis Society and the International Chair on Cardiometabolic Risk Working Group on Visceral Obesity jointly released a statement in “The Lancet: Diabetes & Endocrinology” to the effect that visceral fat is a risk factor for T2DM, atherosclerosis and CVD [28]. However, few studies have focused on the effect of visceral fat area (VFA) on CIMT in patients with T2DM.

In view of the regional and ethnic differences that might impact the distribution of body fat in individuals, our study focused on populations in Western China. The purpose of our study was to investigate the impacts of VFA and other traditional risk factors on CIMT among patients with T2DM. Furthermore, to make the results more reliable, logistic regression analysis and a decision tree model were simultaneously applied.

## Methods

### Subjects

This is a cross-sectional study in which patients with T2DM were recruited from March 2018 to August 2019 in the Department of Endocrinology, the Sixth Affiliated Hospital of Kunming Medical University, Yuxi City, Yunnan Province, China, and the Clinical Medicine Subcenter for National Standardized Metabolic Disease Management Center (MMC). The inclusionwere the diagnostic criteria for T2DM established by the American Diabetes Association (ADA) [29].

The exclusion criteria were as follows: (1) acute complications of diabetes mellitus; (2) malignant tumor; (3) severe liver dysfunction; (4) estimated glomerular filtration rate (eGFR) < 30 mL/min; (5) acute and chronic infectious diseases; (6) positive islet autoantibodies; (7) age ≥ 85 years and age < 18 years; (8) pregnancy; and (9) a history of angina, myocardial infarction, heart failure, abdominal aortic aneurysm, stroke and other cardiovascular diseases or typical clinical manifestations, electrocardiograms and other imaging evidence supporting the diagnosis of these diseases. Finally, a total of 1372 patients with T2DM were included.

### Ethical principles

This study was conducted in line with the principles set out in the Helsinki Declaration and approved by the Ethics Committee of the Sixth Affiliated Hospital of Kunming Medical University (No. 201934). Written informed consent was obtained from all subjects.

### Clinical information collection

The basic clinical information of the patient was recorded in detail, including sex, age, and smoking status. Height and weight were measured in the standing position. Waist circumference was measured at the horizontal plane midway between the lowest rib and the iliac crest. Hip circumference was measured at the widest part of the buttocks, and the waist-hip ratio (WHR) was calculated. Body mass index (BMI) was calculated as the weight (kg) divided by the square of the height (m^2^). After sitting quietly for 5–10 min, heart rate was monitored. Systolic blood pressure (SBP) and diastolic blood pressure (DBP) of the bilateral upper arm were measured twice with a standard cuff mercury sphygmomanometer. The mean values of SBP and DBP were recorded.

### Laboratory examination

All the subjects avoided high-fat food and alcohol 1 day before blood collection. Venous blood was collected the next day on an empty stomach to assess the levels of fasting blood glucose (Glu0), fasting serum C-peptide (C0), fasting serum insulin (Ins0), glycated hemoglobin A1c (HbA1c), triglyceride (TG), cholesterol (TC), high-density lipoprotein cholesterol (HDL-c), low-density lipoprotein cholesterol (LDL-c), serum creatinine (Scr), blood urea nitrogen (BUN), and uric acid (UA). The estimated glomerular filtration rate (eGFR) was calculated according to the “Chinese-abbreviated Modification of Diet in Renal Disease (c-aMDRD)” [30–32]. Thereafter, all subjects underwent an oral glucose tolerance test (OGTT) to subsequently measure the 2-h postprandial blood glucose (Glu120), 2-h postprandial serum C-peptide (C120) and 2-h postprandial serum insulin (Ins120) levels.

The eGFR was calculated as follows: eGFR (mL/min/1.73 m^2^) = 175 × Scr^−1.234^ × age^−0.179^ ×  (0.79 if female) (note that the unit of Scr in the formula is mg/dl).

### Measurement of common carotid intima-media thickness

All subjects were supine and the anterior neck was fully exposed with the head back and inclined to the side away from the ultrasound physician while taking care to avoid muscle tension caused by overextension of the neck. A high-resolution color ultrasound system (iE33, Philips, USA) was used to measure the common carotid intima thickness at the far wall of the bilateral common carotid arteries approximately 1 cm proximal to the carotid bifurcation. Both the left CIMT (L-CIMT) and right CIMT (R-CIMT) were recorded. Measurements of CIMT were performed by experienced ultrasound physicians.

### Measurement of visceral fat area and subcutaneous fat area

The VFA and subcutaneous fat area (SFA) of the subjects in the supine position were determined with a visceral fat analyzer (HDS-2000, Omron, China) using the bioelectrical impedance method. Measurements of VFA and SFA were performed by physicians in the MMC.

### Calculation of the ankle brachial index

All subjects were supine, with arms and legs at the same level as the heart, for a minimum of 10 min before measurement. A blood pressure monitor (BP-203RPE III, Omron, China) was employed to measure the ankle artery pressure and brachial artery pressure. The ankle brachial index (ABI) was calculated as the ankle artery pressure divided by the brachial artery pressure. Both the left ABI (L-ABI) and right CIMT (R-ABI) were recorded. Measurements of ABI were performed by physicians in the MMC.

### Definitions

Based on the “Chinese Guidelines for the Prevention and Treatment of Hypertension” [33] in treated or untreated subjects, SBP ≥ 140 mmHg and/or DBP ≥ 90 mmHg is defined as hypertension.

Based on the “Expert Consensus on Integrated Management of Type 2 Diabetes and Obesity in China” [34], a BMI ≥ 24 kg/m^2^ was defined as overweight, and a BMI ≥ 28 kg/m^2^ was defined as obesity. In addition, abdominal obesity was defined as a waist circumference ≥ 90 cm in male or ≥ 80 cm in female participants.

Based on the “Guidelines for vascular ultrasonography” [35], CIMT ≥ 1.0 mm was defined as increased CIMT.

Based on the “Guidelines for the Prevention and Treatment of Type 2 Diabetes in China” [36], VFA ≥ 80 cm^2^ was defined as increased VFA; ABI ≤ 0.9 was defined as stenosis, and ABI > 0.9 was defined as nonstenosis; Glu0 < 4.4 mmol/L was defined as excessively controlled, 4.4 mmol/L ≤ Glu0 ≤ 7.0 mmol/L was defined as well controlled, and Glu0 > 7.0 mmol/L was defined as poorly controlled; Glu120 ≤ 7.8 mmol/L was defined as well controlled, 7.8 mmol/L < Glu120 < 10 mmol/L was defined as generally controlled, and Glu120 ≥ 10 mmol/L was defined as poorly controlled; and HbA1c < 7% was defined as well controlled, 7% ≤ HbA1c < 8% was defined as generally controlled, and HbA1c ≥ 8% was defined as poorly controlled. The threshold for SFA in the Chinese population has not been clearly defined; therefore, we defined SFA ≥ 100 cm^2^ as “increased SFA” based on a previous study [37].

Based on the “Chinese Guidelines on Prevention and Treatment of Dyslipidemia in Adults” [38], meeting any of the following criteria was defined as dyslipidemia: TC ≥ 6.22 mmol/L; TG ≥ 2.26 mmol/L; or LDL-c ≥ 4.14 mmol/L.

Based on “Kidney Disease Improving Global Outcomes (KDIGO)” [39, 40]: eGFR ≥ 90 mL/min was defined as GFR category 1 (G1), 60 mL/min ≤ eGFR < 90 mL/min was defined as GFR category 2 (G2), and 30 mL/min ≤ eGFR < 60 mL/min was defined as GFR category 3 (G3).

Based on the “Chinese Multidisciplinary Expert Consensus on the Diagnosis and Treatment of Hyperuricemia and Related Diseases” [41], hyperuricemia (HUA) was defined as a fasting serum UA > 420 μmol/L in male subjects and > 360 μmol/L in female subjects.

### Statistical analysis

Continuous variables are presented as the mean ± standard deviation (SD), and categorical variables are presented as percentages (shown in Table 1). On the basis of the CIMT values, subjects were divided into two groups: CIMT < 1.0 mm [CIMT (–) group, n = 965] and CIMT ≥ 1.0 mm [CIMT (+) group, n = 407]. Differences between the two groups were assessed. The continuous variables with normal distributions were analyzed by Welch’s *t* test, and variables with skewed distributions were analyzed by the Mann–Whitney *U* test. For categorical variables, the Chi square test was used. The correlations of VFA with BMI, waist circumference and SFA were analyzed by Pearson correlation analysis. Both logistic regression analysis and decision tree modeling were performed to establish a correlation factor model for CIMT in patients with T2DM. The methods for the logistic regression were based on *forward selection (likelihood ratio)*, with *p *< 0.05 as the entry criterion and *p *> 0.1 as the removal criterion. The decision tree method was based on the *Chi squared automatic interaction detector (CHAID)*. Seventy percent of the subjects were set as the training dataset to build the model, and the remaining thirty percent were set as the test dataset to verify the model.

**Table 1:** Clinical baseline characteristics of patients with type 2 diabetes

Variable	Total N = 1372	CIMT(−) group N = 965	CIMT(+) group N = 407	*P*
Gender (male/female)	843/529 (61.4%, 38.6%)	558/407 (57.8%, 42.2%)	285/122 (70%, 30%)	0.000^a^**
Smoking (no/yes)	809/563 (59.0%, 41.0%)	654/311 (67.8%, 32.2%)	155/252 (38.1%, 61.9%)	0.000^a^**
Age (years)	53.66 ± 11.44	53.04 ± 11.49	55.12 ± 11.20	0.001^b^**
Heart rate (beat/min)	75.51 ± 10.86	75.85 ± 10.94	74.70 ± 10.64	0.042^b^*
SBP (mm/Hg)	126.42 ± 16.28	125.37 ± 16.07	128.90 ± 16.51	0.000^b^**
DBP (mm/Hg)	76.52 ± 9.86	76.05 ± 9.70	77.64 ± 10.16	0.005^b^**
Height (cm)	161.77 ± 9.25	161.42 ± 9.15	162.60 ± 9.44	0.013^b^*
Weight (kg)	67.09 ± 12.64	66.15 ± 12.40	69.32 ± 12.94	0.000^b^**
BMI (kg/m^2^)	25.42 ± 3.60	25.19 ± 3.57	25.98 ± 3.61	0.000^b^**
Waist circumference (cm)	88.74 ± 9.29	87.99 ± 9.22	90.50 ± 9.22	0.000^b^**
Hip circumference (cm)	96.30 ± 7.21	95.95 ± 7.27	97.12 ± 7.01	0.004^b^**
WHR	0.921 ± 0.07	0.92 ± 0.08	0.93 ± 0.07	0.006^b^**
VFA (cm^2^)	86.34 ± 39.82	82.54 ± 38.23	95.34 ± 42.05	0.000^b^**
SFA (cm^2^)	172.54 ± 60.80	168.42 ± 60.26	182.33 ± 61.01	0.000^b^**
Glu0 (mmol/L)	9.55 ± 3.57	9.58 ± 3.76	9.46 ± 3.08	0.678^b^
Glu120 (mmol/L)	14.84 ± 4.98	14.69 ± 4.94	15.20 ± 5.05	0.056^b^
Ins0 (µIU/mL)	9.31 ± 5.90	9.21 ± 5.94	9.56 ± 5.80	0.316^b^
Ins120 (µIU/mL)	24.03 ± 18.78	23.76 ± 18.93	24.67 ± 18.42	0.258^b^
C0 (µg/L)	1.48 ± 1.18	1.45 ± 1.19	1.55 ± 1.17	0.126^b^
C120 (µg/L)	3.41 ± 9.23	3.40 ± 10.61	3.43 ± 4.46	0.023^b^*
HbA1C (%)	9.28 ± 2.39	9.23 ± 2.42	9.39 ± 2.33	0.280^b^
eGFR (mL/min/1.73m^2^)	137.05 ± 112.14	135.57 ± 67.83	140.57 ± 177.56	0.015^b^*
BUN (mmol/L)	5.346 ± 10.84	5.41 ± 12.80	5.21 ± 2.73	0.002^b^**
Scr (µmol/L)	64.18 ± 20.69	63.18 ± 20.06	66.54 ± 21.95	0.002^b^**
UA (µmol/L)	332.75 ± 95.49	329.98 ± 95.37	339.31 ± 95.57	0.022^b^**
TG (mmol/L)	2.72 ± 2.51	2.81 ± 2.66	2.53 ± 2.10	0.436^b^
TC (mmol/L)	4.61 ± 1.33	4.64 ± 1.35	4.54 ± 1.28	0.166^b^
HDL-c (mmol/L)	1.34 ± 4.29	1.40 ± 5.11	1.19 ± 0.40	0.006^b^**
LDL-c (mmol/L)	2.30 ± 0.90	2.29 ± 0.90	2.34 ± 0.88	0.497^b^
L-CIMT (mm)	1.49 ±1.83	0.73 ± 0.13	3.29 ± 2.57	0.000^b^**
R-CIMT (mm)	1.48 ± 1.83	0.72 ± 0.13	3.26 ± 2.71	0.000^b^**
Mean value of CIMT (mm)	1.48 ± 1.84	0.73 ± 0.13	3.27 ± 2.62	0.000^b^**
L-ABI	1.14 ± 0.09	1.14 ± 0.08	1.15 ± 0.09	0.057^b^
R-ABI	1.14 ± 0.09	1.14 ± 0.08	1.15 ± 0.09	0.051^b^
Mean value of ABI	1.14 ± 0.08	1.14 ± 0.08	1.15 ± 0.08	0.055^b^

*SBP* systolic blood pressure, *DBP* diastolic blood pressure, *BMI* body mass index, *WHR* waist hip ratio, *VFA* visceral fat area, *SFA* subcutaneous fat area, *Glu0* fasting blood glucose, *Glu120* 2-hour postprandial blood glucose, *Ins0* fasting serum insulin, *Ins120* 2-hour postprandial serum insulin, *C0* fasting serum C peptide, *C120* 2-hour postprandial serum C-peptide, *HbA1C* glycated hemoglobin A1c, *eGFR* estimated glomerular filtration rate, *BUN* blood urea nitrogen, *Scr* serum creatinine, *UA* uric acid, *TG* triglyceride, *TC* cholesterol, *HDL-c* high density lipoprotein cholesterol, *LDL-c* low density lipoprotein cholesterol, *CIMT* carotid intima media thickness, *L-CIMT* left carotid intima media thickness, *R-CIMT* right carotid intima media thickness, *ABI* ankle brachial index, *L-ABI* left ankle brachial index, *R-ABI* right ankle brachial index

^a^Chi-square test

^b^Mann-Whitney’s *U* test

*P<0.05, **P<0.01

Variable assignments were used in the logistic regression and decision tree modeling. *p* < 0.05 was considered an indicator of a significant difference. All analyses were evaluated with SPSS version 20.0 (SPSS 20.0, IBM, USA). *A power analysis was conducted with PASS 19.0 to* calculate the number of subjects needed in this study; PASS (https://www.ncss.com/software/pass) is a professional software used to calculate sample size [42]. We found that the incidence of increased CIMT in patients with T2DM was 23.5% in a study conducted in a Chinese population [27]. Based on these data, a sample size of 1291 was needed. Thus, the inclusion of 1372 patients in this study is reasonable and provides sufficient statistical power. The calculation result is showed in the Additional file 1.

The quality of the studies was evaluated using the Strengthening the Reporting of Observational Studies in Epidemiology (STROBE) checklist (https://www.strobe-statement.org/index.php?id=available-checklists). The STROBE checklist is shown in the Additional file 2.

## Results

### Clinical baseline characteristics

A total of 1372 patients with T2DM were finally enrolled in the study (61.4% male and 38.6% female; mean age: 53.66 ± 11.44 years). On the basis of CIMT values, the subjects were divided into the CIMT < 1.0 mm group [CIMT (−), n = 965] and the CIMT ≥ 1.0 mm group [CIMT (+), n = 407]. Table 1 summarizes the clinical baseline characteristics of the participants in the two groups (Additional file 3).

### Univariate analysis of possible risk factors for CIMT in patients with T2DM

Univariate analyses showed that the age, height, weight, BMI, waist circumference, hip circumference, WHR, SBP, DBP, VFA, SFA, C120, Scr, and UA in the CIMT (+) group were significantly higher than those in the CIMT (−) group (all *p *< 0.05). In addition, sex, smoking status, heart rate and BUN were significantly different between the two groups (all *p *< 0.05). Other factors, including Glu0, Glu120, Ins0, Ins120, C0, HbA1c, TG, TC, LDL, L-ABI, R-ABI, and the mean value of ABI were not significantly different between the two groups (all *p *> 0.05). These results are shown in Table 1.

### Correlation analysis of VFA with BMI, waist circumference and SFA

**Fig. 1:**
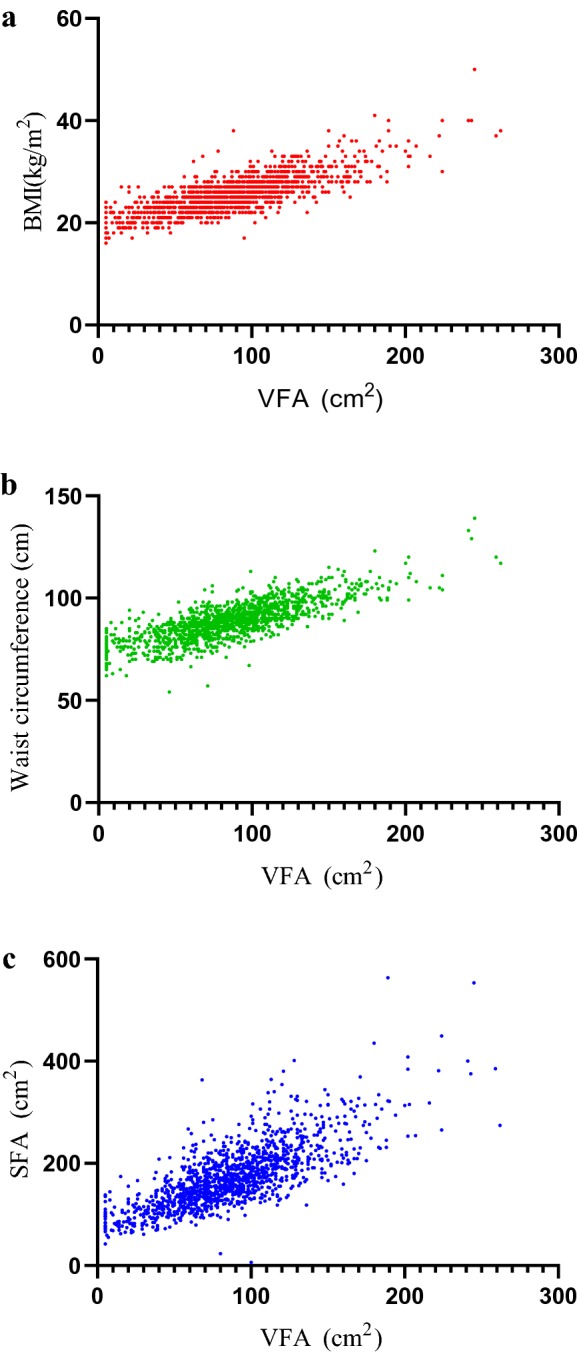
Correlations of VFA with BMI (**a**), Waist circumference (**b**) and SFA (**c**). *VFA* visceral fat area, *BMI* body mass index, *SFA* subcutaneous fat area

BMI, waist circumference and SFA are commonly used in the clinical evaluation of body fat distribution. In the present study, a correlation analysis between VFA and these indicators was conducted. The Pearson correlation coefficients *r* of VFA with BMI, waist circumference and SFA were 0.781, 0.787, and 0.763, respectively. The results are depicted in Table 2 and Fig. 1.

**Table 2:** Correlations of VFA with BMI, waist circumference and SFA

	BMI	Waist circumference	SFA
VFA			
Pearson correlation	0.781^**^	0.787^**^	0.763^**^
*P* (2-tailed)	0.000	0.000	0.000

*VFA* visceral fat area, *BMI* body mass index, *SFA* subcutaneous fat area

**Correlation is significant at the 0.01 level (2-tailed)

### Logistic regression analysis of factors associated with CIMT in patients with T2DM

The variable assignments used in the logistic regression are shown in Table 3. The relevant factors that were ultimately included in the logistic regression model were smoking, sex, VFA and BMI (shown in Table 4). The results showed that the risk of CIMT was 5.759 times higher in smokers than in nonsmokers (odds ratio (OR) = 5.759, 95% confidence interval (CI) (3.884, 8.541)). Patients with VFA ≥ 80 cm^2^ had a 1.364-fold higher risk of CIMT compared with those with VFA < 80 cm^2^ (OR = 1.364, 95% CI (1.018, 1.828)). Compared to male patients, female patients had a 2.239-fold higher risk of CIMT (OR = 2.239, 95% CI (1.486, 3.733)). The larger the BMI value was, the higher the risk of CIMT (OR = 1.186, 95% CI (1.017, 1.384)).

**Table 3:** Variable assignment used in logistic regression and decision tree

Variable	Assignment
CIMT	L-CIMT or R-CIMT met any of the following criteria
	0 = normal (CIMT < 1 mm)
	1 = increased CIMT (CIMT ≥ 1 mm)
Gender	0 = male
	1 = female
Age	1 = young and middle-aged (age < 60 years)
	2 = old age (age ≥ 60 years)
Smoking	0 = no
	1 = yes
Blood pressure	0 = normal (SBP < 140 mmHg and/or DBP < 90 mmHg)
	1 = hypertention (SBP ≥ 140 mmHg and/or DBP ≥ 90 mmHg)
BMI	1 = normal (BMI < 24 kg/m^2^)
	2 = overweight (24 kg/m^2^ ≤ BMI < 28 kg/m^2^)
	3 = obesity (28 kg/m^2^ ≤ BMI < 30 kg/m^2^)
	4 = severe obesity (BMI ≥ 30 kg/m^2^)
Waist circumference	0 = normal (male < 90 cm, female < 85 cm)
	1 = increased waist circumference (male ≥ 90 cm, female ≥ 85 cm)
VFA	0 = normal (VFA < 80 cm^2^)
	1 = increased VFA (VFA ≥ 80 cm^2^)
SFA	0 = normal (SFA < 100 cm^2^)
	1 = increased SFA (SFA ≥ 100 cm^2^)
Glu 0	1 = excessively controlled (Glu0 < 4.4 mmol/L)
	2 = well controlled (4.4 mmol/L ≤ Glu0 ≤ 7.0 mmol/L)
	3 = poorly controlled (Glu0 > 7.0 mmol/L)
Glu120	1 = well controlled (Glu120 ≤ 7.8 mmol/L)
	2 = generally controlled (7.8 mmol/L < Glu120 < 10 mmol/L)
	3 = poorly controlled (Glu120 ≥ 10 mmol/L)
HbA1c	1 = well controlled (HbA1c < 7 %) 2 = generally controlled (7 % ≤ HbA1c < 8 %) 3 = poorly controlled (HbA1c ≥ 8 %)
CKD	1 = G1 (eGFR ≥ 90 ml/min)
	2 = G2 (60 ml/min ≤ eGFR < 90 ml/min)
	3 = G3 (30 ml/min ≤ eGFR < 60 ml/min)
UA	0 = normal
	1 = hyperuricemia (male > 420 ummol/L; female > 360 umol/L)
Blood lipid	0 = normal
	1 = dyslipidaemia (met any of the following criteria: TG ≥ 2.26 mmol/L; TC ≥ 6.26 mmol/L; LDL≥ 4.14 mmol/L; HDL < 1.04 mmol/L)
ABI	0 = non-stenosis (L-ABI and/or R-ABI > 0.9)
	1 = stenosis (L-ABI or R-ABI ≤ 0.9)

*CIMT* carotid intima media thickness, *SBP* systolic blood pressure, *DBP* diastolic blood pressure, *BMI* body mass index, *VFA* visceral fat area, *SFA* subcutaneous fat area, *Glu0* fasting blood glucose, *Glu120* 2-hour postprandial blood glucose, *HbA1C* glycated hemoglobin A1c, *CKD* chronic kidney disease, *eGFR* estimated glomerular filtration rate, *UA* uric acid, *TG* triglyceride, *TC* cholesterol, *LDL-c* low density lipoprotein cholesterol, *HDL-c* high density lipoprotein cholesterol, *ABI* ankle brachial index, *L-ABI* left ankle brachial index, *R-ABI* right ankle brachial index

**Table 4:** Logistic regression analysis of associated risk fators of CIMT in patients with T2DM

Variable	B	S.E,	Wals	*p*	OR	95% C.I.
Gender	0.806	0.209	14.852	0.000	2.239	1.486–3.373
Smoking	1.751	0.201	75.846	0.000	5.759	3.884–8.541
BMI	0.171	0.079	4.724	0.030	1.186	1.017–1.384
VFA	0.311	0.149	4.333	0.037	1.364	1.018–1.828
Constant	− 2.508	0.233	115.636	0.000	0.081	

*BMI* body mass index, *VFA* visceral fat area

### Decision tree modeling of factors associated with CIMT in patients with T2DM

**Fig. 2:**
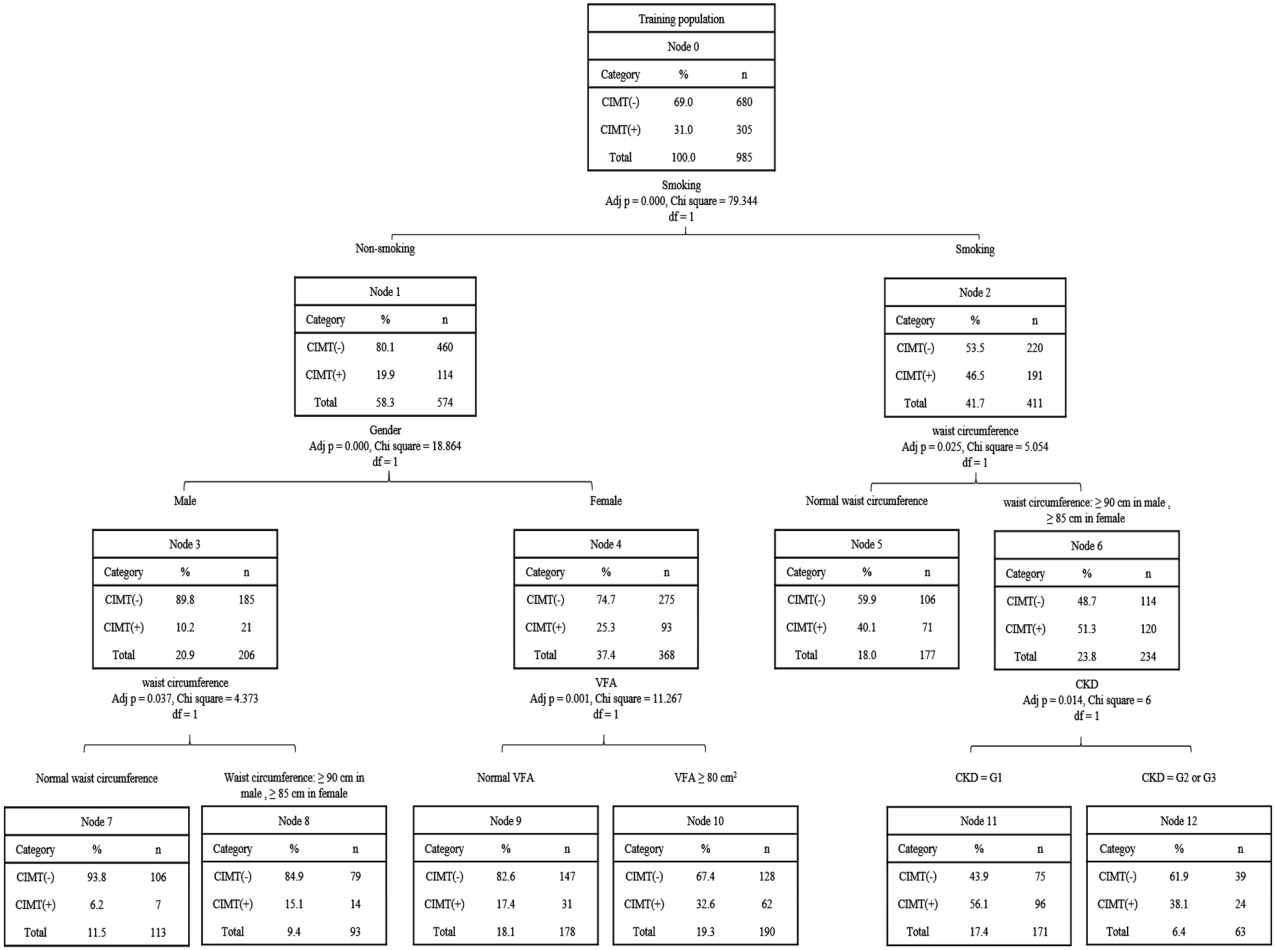
Training dataset of decision tree model. *R rule* CIMT carotid intima media thickness, *VFA* visceral fat area, *CKD* chronic kidney disease

The variables included in the decision tree model are shown in Table 3; these were the same variables as those used in the logistic regression analysis. In the present study, 70% of the subjects were randomly set as a training dataset (n = 985) to build the model, and the remaining 30% were set as a test dataset (n = 387) to verify the model. As described in Fig. 2, smoking was the first variable or root node in this decision tree model, followed by sex, waist circumference, VFA and CKD in order of their importance in the study decision tree. Table 5 summarizes 7 “if–then” rules extracted by tracing a path from the root node to each leaf node.

**Table 5:** Rules extracted from decision tree

Rule	If	Then
R1	If: non-smoking, male and normal waist circumference	Then class: patients with normal CIMT (93.8%)
R2	If: non-smoking, male and waist circumference ≥ 90cm	Then class: patients with increased CIMT (15.1%)
R3	If: non-smoking, female and normal VFA	Then class: patints with normal CIMT (82.6%)
R4	If: non-smoking, female and VFA ≥ 80 cm^2^	Then class: patints with increased CIMT (32.6%)
R5	If: smoking, normal waist circumference	Then class: patients with normal CIMT (59.9%)
R6	If: smoking, waist circumference ≥ 90 cm in male or ≥ 85 cm in female and CKD = G1 (eGFR ≥ 90 ml/min)	Then class: patients with increased CIMT (56.1%)
R7	If: smoking, waist circumference ≥ 90 cm in male or ≥ 85 cm in female and CKD = G2 or G3 (30 ml/min ≤ eGFR < 90 ml/min)	Then class: patients with normal CIMT (61.9%)

*R* rule, *CIMT* carotid intima media thickness, *VFA* visceral fat area, *CKD* chronic kidney disease

## Discussion

The aim of the present study was to provide reliable evidence about the effects of various factors on the risk of CIMT among patients with T2DM in Western China. Unlike most previous studies, in addition to a traditional statistical analysis, we simultaneously employed a decision tree analysis to make our results more reliable.

### The effects of traditional risk factors on CIMT in patients with T2DM

Age, sex, and smoking are well-known traditional risk factors affecting CIMT [16–20]. Our results were basically consistent with those of previous studies. Univariate analyses showed that there were significant differences in sex, age, and smoking status between the CIMT (+) group and the control group. Correspondingly, both the logistic regression and decision tree models showed that smoking was the most crucial risk factor for CIMT in patients with T2DM. Interestingly, several previous studies have shown that in a nondiabetic population, the mean value of CIMT was higher in men than in women [43, 44]. However, a study from Taiwan [18] investigated the factors determining sex differences in CIMT in different age groups. The results showed that although the mean value of CIMT was higher in men than in women, the rapid increase in cardiovascular risk factors in women after 55 years was a vital cause of CIMT thickening in this age group. In the present study, logistic regression showed that being female was a risk factor for CIMT thickening associated with T2DM, which should be considered in the context of the following possible factors: first, the special nature of the study population. The subjects in our study were limited to patients with T2DM, rather than a healthy checkup population. Second, menopause is an undisputed important risk factor for both CVD [45–48] and T2DM [49–51]. The mean age at which Chinese women undergo menopause is generally between 45 and 50 years old [52, 53], and we recruited a total of 529 women with T2DM, of whom more than 80% were over 45 years old, which meant that the vast majority of female patients were in menopausal or postmenopausal states. The subsequent decision tree analysis also showed that nonsmoking women with VFA ≥ 80 cm^2^ had an increased probability of CIMT thickening. Therefore, it would be beneficial to increase the sample size in different age groups in the future to verify the possible influence of the interactions among sex, age and other factors.

We also explored the effect of lipids on CIMT in patients with T2DM. The results showed that the HDL-c level in the CIMT (+) group was significantly lower than that in the CIMT (−) group (*p *< 0.01), while other blood lipid indicators, including TG, TC, and LDL-c, were not different between the two groups (*p *= 0.436, *p *= 0.166, *p *= 0.497, respectively). In addition, several studies have shown that blood pressure [21], blood glucose level [25], blood glucose level fluctuation and C-peptide level [26, 27] were associated with CIMT thickening. In this study, SBP, DBP and C120 were higher in the CIMT (+) group than in the control group (all *p *< 0.01), but there was no significant difference in Glu0, Glu120, C0 and HbA1c between the two groups (all *p *> 0.05). It is worth noting that both the logistic regression model and the decision tree model ultimately excluded the relevant indicators blood glucose, blood lipids and blood pressure from the model. These discrepancies between our study and previous studies might have arisen from the following factors: first, compared with other studies, we chose CIMT ≥ 1.0 mm as the threshold for CIMT thickening [35], which was stricter and more in line with the characteristics of the Chinese population; moreover, the design, study population, and exclusion criteria among the studies were distinct; last, the use of antidiabetic, antihypertensive and lipid-lowering drugs may have influenced the results of the study to some extent.

### Effect of VFA on CIMT in patients with T2DM

In recent years, the global prevalence of obesity has markedly increased, and the incidence of obesity-related T2DM has increased annually [54]. Unlike in Caucasian individuals, body fat in Chinese individuals tends to accumulate in the abdominal cavity, which is more likely to result in abdominal obesity. A study based on the Chinese population showed that abdominal obesity is currently an increasing trend in China, with an adult prevalence rate of 29.1% (28.6% for men and 29.6% for women) [55]. More importantly, the prevalence rates of T2DM in the overweight and obese population in China are 12.8% and 18.5%, respectively [56]. Therefore, it is very important to explore the internal relationships among fat metabolism, T2DM and vascular complications.

Increasing evidence has shown that visceral fat is a risk factor for T2DM, atherosclerosis and cardiovascular and cerebrovascular diseases [28, 57]. There are also racial and geographical differences with regard to the definition of VFA thresholds. VFA ≥ 100 cm^2^ is generally accepted as increased visceral fat in Europe, the United States and Japan [58, 59]. Geographically and ethnically, although China and Japan are very similar, VFA ≥ 80 cm^2^ is recommended as the optimal threshold for evaluating abdominal obesity patients with T2DM in the Chinese population [36, 60]. Therefore, in this study, VFA ≥ 80 cm^2^ was used as the standard to evaluate the increase in visceral fat. The VFA measurement method in our study was bioelectrical impedance, which has a good correlation with CT [61]. Previous studies have illustrated that VFA is a crucial risk factor for CIMT thickening in middle-aged and elderly Chinese populations [60, 62]. Univariate analyses in the present study showed that in patients with T2DM, VFA was higher in the CIMT (+) group than in the CIMT (−) group (*p *< 0.01). Furthermore, both the logistic regression analysis and decision tree model emphasized that VFA was a risk factor for CIMT thickening in patients with T2DM.

### The influence of traditional obesity-related factors on CIMT in patients with T2DM

With consideration of the metabolic characteristics of the Chinese population, we defined BMI ≥ 24 kg/m^2^ as overweight and BMI ≥ 28 kg/m^2^ as obesity; we defined waist circumference ≥ 90 cm in males and waist circumference ≥ 80 cm in females as abdominal obesity [63]. The univariate analyses showed that BMI, waist circumference, hip circumference and WHR were significantly higher in the CIMT (+) group than in CIMT (−) group (all *p* < 0.01). The correlation between VFA and waist circumference was relatively stronger (Pearson correlation coefficient r = 0.787) than the correlation between VFA and BMI. However, it was noteworthy that, unlike VFA, neither BMI nor waist circumference was included in the logistic regression analysis and decision tree at the same time. On the one hand, BMI cannot accurately measure the change in abdominal fat distribution among individuals [64] and the guidelines from the ACC/AHA on the assessment of cardiovascular risk [65] does not consider BMI to be a predictor of cardiovascular risk. In addition, a small group of people in the obese or overweight population never have CVD, leading to the proposed concept of “metabolically healthy obesity” (MHO), which is closely associated with younger age, more aerobic exercise, comprehensive and balanced nutritional status and less VFA [66]. Likewise, although a reduction in waist circumference is always associated with a reduction in visceral adipose tissue (VAT), waist circumference does not precisely reflect changes in VFA [28].

Previous research from Wang et al. [60] found that CIMT increased as VFA increased in both the BMI < 25 kg/m^2^ group and the BMI ≥ 25 kg/m^2^ group in non-T2DM Chinese participants. Other studies based on Japanese T2DM populations have shown that subjects with normal BMI and increased VFA (BMI < 25 kg/m^2^ and VFA ≥ 100 cm^2^ group) were more likely to suffer from systemic arteriosclerosis [67] and arterial stiffness [68].

In our study, a similar subgroup analysis was conducted. Taking into account the metabolic characteristics of the Chinese population, the subjects in our study were divided into four subgroups according to VFA and BMI: VFA < 80 cm^2^ and BMI < 24 kg/m^2^ [VFA (−) BMI (−) group, n = 386], VFA < 80 cm^2^ and BMI ≥ 24 kg/m^2^ [VFA (−) BMI (+) group, n = 230], VFA ≥ 80 cm^2^ and BMI < 24 kg/m^2^ [VFA (+) BMI (−) group, n = 104], and VFA ≥ 80 cm^2^ and BMI ≥ 24 kg/m^2^ [VFA (+) BMI (+) group, n = 652]. However, there were no significant differences in CIMT among the four groups (all *p* > 0.05). These results are illustrated in the Additional file 1. Differences in the exclusion criteria and VFA threshold values may be the reason for the divergence between the results of this study and other studies.

In addition to BMI and waist circumference, SFA is also an indicator that can be used to evaluate body fat distribution. Visceral and ectopic fats are deposited when the subcutaneous adipose tissue (SAT) cannot expand to accommodate excessive energy intake [69]. Nevertheless, SFA and VFA have different anatomical and functional significance [69, 70]. The results of previous studies regarding the relationship between SFA and CVD are controversial [71–73]. Some studies have suggested that increased SFA was positively associated with CVD, while some studies have suggested that moderately increased SFA is beneficial for the balance of blood glucose and blood lipids, thereby exerting protective effects on cardiovascular health.

Although the SFA in the CIMT (+) group was significantly greater than that in the CIMT (−) group in this study, and the correlation analysis between VFA and SFA showed that the Pearson correlation coefficient r was 0.762, the logistic regression and decision tree models excluded SFA from the model. A study from Japan showed that increased VFA (≥ 100 cm^2^) and low SFA (≤ 100 cm^2^) were the determinants of atherosclerosis in T2DM patients [37]. Unfortunately, only 5 patients from the total sample of subjects in our study had the characteristics of SFA < 100 cm^2^ and VFA ≥ 80 cm^2^, so a valuable subgroup analysis could not be performed. It is necessary to expand the sample size of this subgroup in the future.

### Decision tree analysis of factors influencing CIMT in patients with T2DM

Although traditional statistical methods such as logistic regression can identify statistically significant risk factors, it is impossible to make qualitative judgments about the likelihood that each risk factor will be at specific levels. A decision tree is an effective machine learning method used to classify data based on a series of rules [74]. It has a “flowchart-like” structure that can extract classification rules from a set of irregular cases, compare the attribute values in each internal node, judge the branches below the node, and obtain the classification conclusion in the leaf nodes. In other words, the paths from root to leaf represent classification rules. Recently, the application of decision trees has become increasingly extensive in the field of medical research [74, 75]. Therefore, to make our results more convincing and robust, we further carried out a decision tree analysis based on the “*CHAID*” method, which has become one of the most common decision tree algorithms. Until now, no research has employed a decision tree to analyze the effect of associated factors on CIMT in patients with T2DM.

As described in Fig. 2, the variables that finally remained in the model included smoking, sex, waist circumference, VFA and CKD. The “if–then” rules summarized in Table 5 were the “details” that were extracted from the decision tree. Specifically, smoking was the most crucial risk factor affecting CIMT in patients with T2DM; among nonsmokers, females were at relatively higher risk for increased CIMT, especially those an increased VFA (VFA ≥ 80 cm^2^). For smokers, an increase in waist circumference (male ≥ 90 cm and female ≥ 85 cm) was a risk factor for increased CIMT, especially in patients with CKD 1 and 2. More importantly, we found that smoking, sex and VFA were essential factors influencing CIMT in patients with T2DM, which was basically consistent with the results of the logistic regression analysis.

### Limitations

In brief, this study was the first to analyze the factors influencing CIMT in patients with T2DM in Western China. However, it should be noted that there were several limitations. First, the present study did not record the use of antidiabetic, lipid-lowering and antihypertensive medications in patients, so it is impossible to exclude the possibility of an influence of these medications. Moreover, we should note that the duration of diabetes mellitus was not available for analysis in this study. Therefore, these factors may have contributed to differences in research findings.

## Conclusions

In the present study, the risk factors for CIMT thickening in patients with T2DM in Western China were explored for the first time. Despite some limitations, there were several valuable findings. First, traditional risk factors such as smoking and increased BMI had significant effects on increased CIMT associated with T2DM. Second, unlike other studies conducted in the population receiving regular health checkups, the data from this study supported female sex as a vital factor influencing CIMT in patients with T2DM. More importantly, our data emphasize the role of VFA in the management and evaluation of diabetic macroangiopathy in the future. Meanwhile, unlike traditional statistical methods, the decision tree model used in our study, with its simple, intuitive and practical hierarchical methods, could help clinicians make more effective risk-based decisions, which is worth promoting in future medical research. In the future, we will continue to expand the sample size, add new indicators to assess vascular disease, and further enrich and strengthen our theory in order to better prevent and manage diabetic vascular disease.

## Supplementary information

**Additional file 1**. Results of sample size calculation.

**Additional file 2**. STROBE Statement—checklist of items that should be included in reports of cross-sectional studies.

**Additional file 3.** Characteristics of patients with type 2 daibetes in subgroups.

## Data Availability

The datasets used in the present study are available from the corresponding author on reasonable request.

## Abbreviations

CVD: cardiovascular disease
T2DM: type 2 diabetes
CIMT: carotid intima media thickness
VFA: visceral fat area
SBP: systolic blood pressure
DBP: diastolic blood pressure
BMI: body mass index
CKD: chronic kidney disease
IDF: International Diabetes Federation
MMC: Metabolic Disease Management Center
WHO: World Health Organization
GFR: glomerular filtration rate
WHR: waist-hip ratio
Glu0: fasting blood glucose
C0: fasting serum C peptide
Ins0: fasting serum insulin
HbA1c: glycated hemoglobin A1c
TG: triglyceride
TC: cholesterol
HDL-c: high-density lipoprotein cholesterol
LDL-c: low-density lipoprotein cholesterol
Scr: serum creatine
BUN: blood urea nitrogen
UA: uric acid
c-aMDRD: Chinese-abbreviated modification of diet in renal disease
OGTT: oral glucose tolerance test
Glu120: 2-h postprandial blood glucose
C120: 2-h postprandial serum C-peptide
Ins120: 2-h postprandial serum insulin
L-CIMT: left CIMT
R-CIMT: right CIMT
SFA: subcutaneous fat area
ABI: ankle brachial index
L-ABI: left ABI
R-ABI: right ABI
KDIGO: Kidney Disease Improving Global Outcomes
G: GFR category
HUA: hyperuricemia
SD: standard deviation
CHAID: Chi squared automatic interaction detector
SPSS: Statistical Package for Social Sciences
