# Supplementary material for: Analysis of risk factors for carotid intima-media thickness in patients with type 2 diabetes mellitus in Western China assessed by logistic regression combined with a decision tree model
Source: Diabetol Metab Syndr. 2020 Jan 28;12:8. doi: 10.1186/s13098-020-0517-8 (PMC6988356; doi:10.1186/s13098-020-0517-8)
Supplement: Supplementary file 1 — Additional file 1. Results of sample size calculation. [file 13098_2020_517_MOESM1_ESM.rtf]

	
Results of sample size calculation 

Confidence Intervals for One Proportion - New
Numeric Results for Two-Sided Confidence Intervals for One Proportion
Confidence Interval Formula: Exact (Clopper-Pearson)

	Sample						
Confidence	Size	Target	Actual	Proportion	Lower	Upper	Width if
Level	(N)	Width	Width	(P)	Limit	Limit	P = 0.5
0.950	1291	0.047	0.047	0.235	0.212	0.259	0.055

References
Fleiss, J. L., Levin, B., Paik, M.C. 2003. Statistical Methods for Rates and Proportions. Third Edition. John
   Wiley & Sons. New York.
Newcombe, R. G. 1998. 'Two-Sided Confidence Intervals for the Single Proportion: Comparison of Seven Methods.'
   Statistics in Medicine, 17, pp. 857-872.

Report Definitions
Confidence level is the proportion of confidence intervals (constructed with this same confidence level,
   sample size, etc.) that would contain the population proportion.
N is the size of the sample drawn from the population.
Width is the distance from the lower limit to the upper limit.
Target Width is the value of the width that is entered into the procedure.
Actual Width is the value of the width that is obtained from the procedure.
Proportion (P) is the assumed sample proportion.
Lower Limit is the lower limit of the confidence interval.
Upper Limit is the upper limit of the confidence interval.
Width if P = 0.5 is the maximum width for a confidence interval with sample size N.

Summary Statements
A sample size of 1291 produces a two-sided 95% confidence interval with a width equal to 0.047
when the sample proportion is 0.235.


	2019/12/25 16:25:38      2

Confidence Intervals for One Proportion - New
Chart Section
