# Supplementary material for: Analysis of risk factors for carotid intima-media thickness in patients with type 2 diabetes mellitus in Western China assessed by logistic regression combined with a decision tree model
Source: Diabetol Metab Syndr. 2020 Jan 28;12:8. doi: 10.1186/s13098-020-0517-8 (PMC6988356; doi:10.1186/s13098-020-0517-8)
Supplement: Supplementary file 2 — Additional file 2. STROBE Statement—checklist of items that should be included in reports of cross-sectional studies. [file 13098_2020_517_MOESM2_ESM.doc]

STROBE Statement—Checklist of items that should be included in reports of ***cross-sectional studies***

|  | Item No | Recommendation |  |  |  |
| --- | --- | --- | --- | --- | --- |
| **Title and abstract** | 1 | (*a*) Indicate the study’s design with a commonly used term in the title or the abstract |  |  |  |
|  |  | (*b*) Provide in the abstract an informative and balanced summary of what was done and what was found | Abstract  Lines 19-50 |  |  |
| Introduction | | |  |  |  |
| Background/rationale | 2 | Explain the scientific background and rationale for the investigation being reported | Backgroud  Lines 52-83 |  |  |
| Objectives | 3 | State specific objectives, including any prespecified hypotheses | Backgroud  Lines 84-90 |  |  |
| Methods | | |  |  |  |
| Study design | 4 | Present key elements of study design early in the paper | Methods  Line 94 |  |  |
| Setting | 5 | Describe the setting, locations, and relevant dates, including periods of recruitment, exposure, follow-up, and data collection | Methods  Subjects  Lines 95-98 |  |  |
| Participants | 6 | (*a*) Give the eligibility criteria, and the sources and methods of selection of participants | Methods  Subjects  Lines 98-100 |  |  |
| Variables | 7 | Clearly define all outcomes, exposures, predictors, potential confounders, and effect modifiers. Give diagnostic criteria, if applicable | Methods  Lines 169-200 |  |  |
| Data sources/ measurement | 8* | For each variable of interest, give sources of data and details of methods of assessment (measurement). Describe comparability of assessment methods if there is more than one group | Methods  Lines 117-166 |  |  |
| Bias | 9 | Describe any efforts to address potential sources of bias | Methods  Lines 101-110 |  |  |
| Study size | 10 | Explain how the study size was arrived at | Methods  Statistical analysis  Lines 221-227 |  |  |
| Quantitative variables | 11 | Explain how quantitative variables were handled in the analyses. If applicable, describe which groupings were chosen and why | Methods  Statistical analysis  Lines 204-206 |  |  |
| Statistical methods | 12 | (*a*) Describe all statistical methods, including those used to control for confounding | Methods  Statistical analysis  Lines 202-221 |  |  |
|  |  | (*b*) Describe any methods used to examine subgroups and interactions | Not applicable |  |  |
|  |  | (*c*) Explain how missing data were addressed | Not applicable.There was no missing data in this study |  |  |
|  |  | (*d*) If applicable, describe analytical methods taking account of sampling strategy | Methods  Statistical analysis  Lines 221-227 |  |  |
|  |  | (*e*) Describe any sensitivity analyses | Not applicable |  |  |
| Results | | |  |  |  |
| Participants | 13* | (a) Report numbers of individuals at each stage of study—eg numbers potentially eligible, examined for eligibility, confirmed eligible, included in the study, completing follow-up, and analysed | Results  Table1  Lines 235-240 |  |  |
|  |  | (b) Give reasons for non-participation at each stage | Not applicable |  |  |
|  |  | (c) Consider use of a flow diagram | Not applicable |  |  |
| Descriptive data | 14* | (a) Give characteristics of study participants (eg demographic, clinical, social) and information on exposures and potential confounders | Results  Table1  Lines 242-249 |  |  |
|  |  | (b) Indicate number of participants with missing data for each variable of interest | Not applicable |  |  |
| Outcome data | 15* | Report numbers of outcome events or summary measures | Results  Table1-5  Figure1-2 |  |  |
| Main results | 16 | (*a*) Give unadjusted estimates and, if applicable, confounder-adjusted estimates and their precision (eg, 95% confidence interval). Make clear which confounders were adjusted for and why they were included | Results  Lines 235-280  Table1-4 |  |  |
|  |  | (*b*) Report category boundaries when continuous variables were categorized | Not applicable |  |  |
|  |  | (*c*) If relevant, consider translating estimates of relative risk into absolute risk for a meaningful time period | Not applicable |  |  |
| Other analyses | 17 | Report other analyses done—eg analyses of subgroups and interactions, and sensitivity analyses | Not applicable |  |  |
| Discussion | | |  |  |  |
| Key results | 18 | Summarise key results with reference to study objectives | Conclusion  Lines 451-460 |  |  |
| Limitations | 19 | Discuss limitations of the study, taking into account sources of potential bias or imprecision. Discuss both direction and magnitude of any potential bias | Limitations  Lines 442-449 |  |  |
| Interpretation | 20 | Give a cautious overall interpretation of results considering objectives, limitations, multiplicity of analyses, results from similar studies, and other relevant evidence | Discussion  Lines 282-440 |  |  |
| Generalisability | 21 | Discuss the generalisability (external validity) of the study results | Conclusion  Lines 460-464 |  |  |
| Other information | | |  |  |  |
| Funding | 22 | Give the source of funding and the role of the funders for the present study and, if applicable, for the original study on which the present article is based | Fundings  Lines 526-531 |  |  |

*Give information separately for exposed and unexposed groups.

**Note:** An Explanation and Elaboration article discusses each checklist item and gives methodological background and published examples of transparent reporting. The STROBE checklist is best used in conjunction with this article (freely available on the Web sites of PLoS Medicine at http://www.plosmedicine.org/, Annals of Internal Medicine at http://www.annals.org/, and Epidemiology at http://www.epidem.com/). Information on the STROBE Initiative is available at www.strobe-statement.org.
