# Supplementary material for: Analysis of risk factors for carotid intima-media thickness in patients with type 2 diabetes mellitus in Western China assessed by logistic regression combined with a decision tree model
Source: Diabetol Metab Syndr. 2020 Jan 28;12:8. doi: 10.1186/s13098-020-0517-8 (PMC6988356; doi:10.1186/s13098-020-0517-8)
Supplement: Supplementary file 3 — Additional file 3. Characteristics of patients with type 2 daibetes in subgroups. [file 13098_2020_517_MOESM3_ESM.docx]

**Additional file**

**characteristics of patients with type 2 daibetes in subgroups**

| **Variable** | **VFA(−) BMI(−)** | **VFA(−) BMI(+)** | **VFA(+) BMI(−)** | **VFA(+) BMI(+)** | ***P* ^##^** |
| --- | --- | --- | --- | --- | --- |
|  | N= 386 | N = 230 | N = 104 | N = 652 |  |
| L-CIMT (mm) | 1.47 ± 1.83 | 1.41 ± 1.76 | 1.22 ± 1.46 | 1.57 ± 1.90 | > 0.05 |
| R-CIMT (mm) | 1.47 ± 1.83 | 1.40 ± 1.83 | 1.24 ± 1.54 | 1.55 ± 1.97 | > 0.05 |
| Mean value of CIMT (mm) | 1.47 ± 1.83 | 1.40 ± 1.78 | 1.23 ± 1.50 | 1.56 ± 1.92 | > 0.05 |

^##^ One-way ANOVA,

Abbreviation: CIMT carotid intima media thickness; L-CIMT left carotid intima media thickness; R-CIMT right carotid intima media thickness
